# A Semantic Surprise in Colon: *Gastrodiscoides hominis*

**DOI:** 10.14309/crj.0000000000001699

**Published:** 2025-05-02

**Authors:** Mathew Vadukoot Lazar, George Sarin Zacharia, Krishnanmurthy Subbian, Jibu Thomas

**Affiliations:** 1 Lifecare Hospital, Mussafah, Abu Dhabi, UAE; 2 Karunya Institute of Technology and Sciences, Tamil Nadu, India; 3 BronxCare Health System, Bronx, New York, USA; 4 Burjeel Medical City, Abu Dhabi, UAE

**Keywords:** gastrodiscoidiasis, *Gastrodiscoides hominis*, colonoscopy, colon flukes, mebendazole

## Abstract

Human gastrodiscoidiasis is a rare, geographically restricted trematode infection caused by *Gastrodiscoides hominis*, an amphistome platyhelminth, also known as the colon fluke. The disease is endemic to Assam in northeastern India, although sporadic cases have been reported in other Asian countries and across different continents. The disease primarily affects the right colon, leading to epithelial desquamation, inflammation, and increased secretion of colonic mucus. We report the case of a 30-year-old Indian man with a history of cramping lower abdominal pain, increased stool frequency, and weight loss, with an inflammatory pattern on routine stool analysis, detected to have multiple platyhelminths adherent to the cecum and ascending colon, subsequently confirmed microbiologically as *G. hominis* and effectively treated with mebendazole. Our case highlights the importance of considering rare helminthic infections in patients with chronic gastrointestinal symptoms, particularly those from endemic areas, and the role of colonoscopy in confirming the diagnosis and guiding treatment.

## INTRODUCTION

Human gastrodiscoidiasis is an exceedingly rare and often geographically localized trematode infection. Caused by the amphistome platyhelminth, *Gastrodiscoides hominis*, the infection was initially described in 1876 in northeastern India, Assam.^1^ Until now, Assam remains the only endemic zone for this rare infection, with a reported prevalence of up to 40%.^2^ Sporadic cases have been reported in other Asian countries, such as Myanmar, Vietnam, Philippines, Pakistan, Thailand, Nepal, and China. Cases have been reported from Nigeria and Guyana, likely related to human migration.^3,4^ The platyhelminth typically resides in the cecum and hence is referred to as the colon fluke. It is a zoonotic, food-borne pathogen; humans are accidental hosts, whereas pigs are the natural hosts. *Helicorbis coenosus*, a mollusc often found abundant adjacent to pigsties, is believed to be the intermediate host.^4^ Symptomatic infection is more frequently reported among children, and deaths have been attributed to this trematode infection in Assam.^1^

## CASE REPORT

A 30-year-old Indian man with no significant history presented with cramping lower abdominal discomfort and an increased stool frequency of 4 months' duration associated with loss of weight and appetite. He denied any overt gastrointestinal bleeding, fever, jaundice, or altered mental status, nor did he have any family history of gastrointestinal neoplasm. Over the past few months, he was evaluated and treated with metronidazole, ciprofloxacin, and loperamide on different occasions, with no fruitful response. The physical examination was unremarkable, except for mild tenderness in the right iliac fossa upon palpation. No abdominal mass, palpable organomegaly, or free fluid was appreciated. His hemogram, biochemical, and thyroid panel were normal except for mild eosinophilia (Table 1). Stool wet mount and trichrome stain microscopy revealed the presence of leucocytes and erythrocytes but no parasites, ova, or cysts. Fecal calprotectin and lactoferrin were positive, suggesting inflammatory diarrhea, whereas the qualitative fat assay was reported to be negative.

**Table 1.** Summary of the laboratory workup

Parameter	Results	Reference range
(A) Specimen: blood/serum		
Hemoglobin (gm/dL)	14.3	14–17.5
Leukocyte count (cells/μL)	7,360	4,400–11,300
Eosinophil count (cells/μL)	660	50–450
Platelet count (×103 cells/μL)	178	150–450
Bilirubin total/direct (μmol/L)	16.2/2.9	0–21/0–5
AST/ALT (IU/L)	24/17	5–40
ALP (IU/L)	227	40–129
Amylase (IU/L)	66	30–110
Prothrombin time (s)	11.5`	9.1–12.1
CRP (mg/L)	<5	0–5
BUN (mmol/L)	6.9	2.14–7.14
Creatinine (μmol/L)	84	62–106
Sodium (mEq/L)	143	135–145
Potassium (mEq/L)	3.6	3.5–5.0
TSH (mIU/L)	2.32	0.4–4
(B) Specimen: stool		
Microscopy		
WBC	Present	Negative
RBC	Present	Negative
Ova & parasites	Not detected	Negative
Calprotectin (mcg/g)	78	<50
Lactoferrin (µg/g)	8.5	<7.25
Fat, qualitative	Negative	Negative

ALT, alanine aminotransferase; ALP, alkaline phosphatase; AST, aspartate aminotransferase; BUN, blood urea nitrate; CRP, C-reacrive protien; RBC, red blood cells; TSH, thyroid stimulating hormone; WBC, white blood cells.

**Figure 1.**
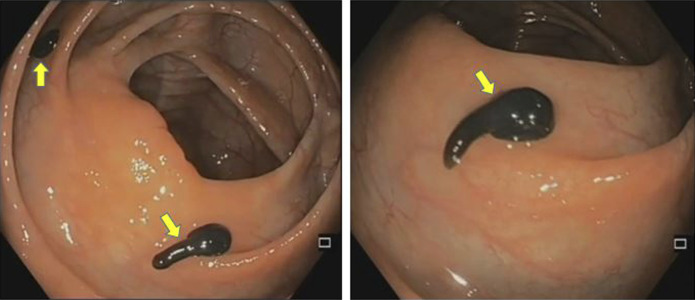
Colonoscopy images revealing multiple greenish-black, conical helminths, each around 10–12 mm long, with their wide circular portion adherent to the mucosa and a narrow free conical end.

Given persistent symptoms and positive stool inflammatory markers, he was scheduled for a colonoscopy for mucosal assessment. Colonoscopy identified multiple greenish-black, conical motile helminths adherent to the mucosa in the cecum and ascending colon. Approximately 15 parasites were identified, each measuring 10–12 mm in length, with a wide, circular portion adherent to the mucosa and a narrow, free, conical end (Figure 1). The parasites were extracted using through-the-scope baskets and sent for microbiology-pathology analysis. The colonic mucosa in the region appeared edematous, with excess luminal mucus. Mucosal bleed was noted after the extraction of helminths, which resolved by itself over a few minutes. The patient tolerated the procedure without complications and had an uneventful short hospital stay.

**Figure 2.**
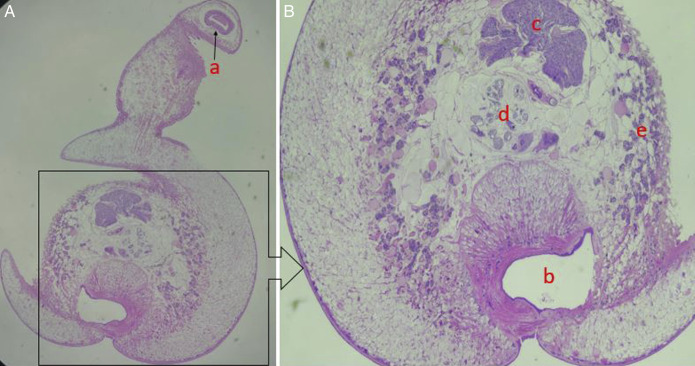
Photomicrographs of *Gastrodiscoides hominis* (40×). (A) Adult fluke with a fleshy pyramidal-shaped body. (a): Anterior muscular oral sucker. (B) The posterior circular discoidal portion of the worm with ventral sucker (b), testis (c), gravid uterus with eggs (d), and vitelline glands (e).

Microscopic analysis reported pyramid-shaped trematodes with a chitinous wall, a prominent ventral sucker at the posterior end, and a tapering anterior end. The ovary and testis are recognized within each helminth, located in the posterior region, with clusters of developing rhomboid eggs (Figure 2). The helminth was reported as *G. hominis*, a platyhelminth, also known as colon fluke. He was administered a dose of mebendazole, 100 mg twice daily, for 3 days, even before the pathogen was reported. The patient reported a complete resolution of symptoms in a week and remained asymptomatic for 6 months. He was provided with a detailed education regarding hand and food hygiene, the importance of avoiding raw food materials, and the proper boiling or cooking of food, as well as other sanitary measures, by our institutional healthcare workers.

## DISCUSSION

*G. hominis* is symmetrical, dorsoventrally flattened, pyramidal-shaped, hermaphrodite, amphistome trematode, with a ventral sucker toward its distal end. It causes gastrodiscoidiasis, most frequently affecting pigs and accidentally involves humans. The affected vertebrates constitute the definitive host, whereas freshwater molluscs are intermediate hosts. The adult helminths inhabit the mammalian cecum, or ascending colon, where they are attached to the mucosa and release eggs. These eggs are flushed down the large intestine and expelled through the mammalian fecal matter. In freshwater, the eggs hatch, releasing a miracidium, a free-swimming larval form that infects the intermediate host. Within the molluscan host, the larva evolves into rediae and cercariae. The cercariae released from the snails are mobile and attach to aquatic vegetation or infect freshwater animals, such as fish or frogs, where they encyst to form metacercariae. The consumption of raw or undercooked aquatic vegetation, freshwater fish, or crustaceans culminates in the infection of definitive hosts, including humans.^3,5^

Poverty, school-age children, consumption of raw vegetables and untreated water, contact with livestock, and poor sanitation have been linked to gastrodiscoidiasis. Humans are accidentally infected after consuming raw or undercooked aquatic vegetation or animals contaminated with metacercariae. The adult helminths attach to the colonic mucosa, causing epithelial desquamation and localized inflammation. Colonic mucus hypersecretion has been reported as well. Heavy infestation results in diarrhea, abdominal pain, malnutrition, and anemia.^6^ Cases of death in children have been linked with gastrodiscoidiasis in endemic regions of north India.^1,3^ Diagnosis relies on the demonstration of the eggs on stool microscopy. The eggs measure about 150 × 70 mm and are operculated and nonembryonated.^5^ The diagnostic accuracy of stool microscopy is highly dependent on the expertise of the operator, which is often limited due to the rarity and geographically isolated nature of the infestation. Adult worms can be visualized during colonoscopy and may be extracted for microbiological and pathological analysis, confirming the diagnosis. In some rare instances, trematodes were also identified during esophagogastroduodenoscopy.^4^

Praziquantel, 25 mg/kg, is considered the drug of choice. A dose of mebendazole, 500 mg administered orally, is equally efficient in treatment. Another alternative is oral tetrachloroethylene, administered at a dose of 0.1 mg/kg on an empty stomach.^5^ Human infection is preventable with improved sanitation, avoiding raw food, and proper cooking techniques. Preventing domesticated pigs from accessing freshwater bodies, ensuring the adequate and hygienic disposal of pig waste without contaminating water sources, conducting periodic parasitological assessments, managing swine effectively, and using molluscicides will help prevent the dissemination of infections. The global human migration and export of aquatic flora and fauna increase the risk of disseminating infections to nonendemic regions. Proper screening of food materials and prompt detection and treatment of infected individuals help to curtail the global dissemination of the disease.^7^

Our patient, a blue-collar worker from India, presented with a protracted course of abdominal discomfort and altered bowel habits, with poor response to empirical medications. He was diagnosed as having a helminthic infection at colonoscopy, which was subsequently confirmed by the microbiology-pathology team as *G. hominis*. No one in our gastroenterology team has ever encountered these helminths in their practice, and it had required almost a week to confirm the microbiological diagnosis. The patient empirically was administered mebendazole before the diagnosis was confirmed; however, the course was efficient in managing his symptoms. Gupte et al documented that spraying hypertonic saline on the parasites promptly results in shrinkage and discoloration.^8^ Hence, spraying hypertonic saline might aid in extracting the parasites with greater ease. However, we had not tried this approach as we were unaware of this response during the colonoscopy.

The case highlights the importance of heightened awareness of rare diseases in the current era of accelerated human migration, the limitations of stool microscopic analysis, and the role of modern endoscopy in diagnosing gastrointestinal parasites. Stool microscopy, although an invaluable tool in the diagnostic algorithm for altered bowel habits, is limited by issues related to the sample, pathogen, technique, and operator expertise. Inadequate sample size, intermittent ovum excretion, low parasite load, improper sample handling, and delayed analysis all contribute to poor sensitivity and false-negative results. It requires significant operator expertise to interpret stool samples, especially for rare pathogens outside the endemic areas, for an accurate diagnosis.

In addition, comprehensive stool microscopy is labor-intensive and time-consuming when the load of ova, cysts, or parasites is low in the stool specimen. Analysis of multiple samples, larger sample sizes, concentration techniques, antigens, and molecular tests may enhance the diagnostic accuracy of stool assays. In our case, the low parasite or ova load, limited operator expertise, and possibly suboptimal sample handling might have resulted in a negative stool result. Last but not least, this case serves as a reminder of the dictum that in any patient with unexplained symptoms and eosinophilia, the possibility of a parasitic etiology needs to be excluded.

Gastrodiscoidiasis, caused by *G. hominis*, is an uncommon zoonotic trematode infection with endemicity primarily in northeastern India, and sporadic reports have been made from other regions. The diagnostic yield of stool microscopy is typically limited outside the endemic areas, necessitating colonoscopy for direct parasite visualization and extraction with subsequent microbiological or histopathological confirmation. Effective treatment includes praziquantel or mebendazole, both of which demonstrate high efficacy in eradicating the parasite and resolving clinical manifestations. Preventive strategies should focus on improving sanitation, restricting pig access to freshwater sources, and controlling intermediate host populations to mitigate further transmission. Enhanced awareness of this rare infection enables endoscopists to identify the helminth during colonoscopy, facilitating the prompt initiation of therapy.

## DISCLOSURES

Author contributions: M. Vadukoot Lazar: acquisition, analysis, or interpretation of data. Reviewed the manuscript. Approved the final version. Agrees to be accountable for all aspects of the work; GS Zacharia: conception of the work, drafting of the manuscript, approval of the final version, agrees to be accountable for all aspects of the work; K. Subbian: acquisition, analysis, or interpretation of data. Reviewed the manuscript. Approved the final version. Agrees to be accountable for all aspects of the work; J. Thomas: interpretation of data, review of the manuscript, approved the final version, and accountable for all aspects of the work. Mathew vadukoot lazar js the article guarantor.

Acknowledgments: We acknowledge all the Lifecare Hospital Gastroenterology Lab staff members, especially Mr. Sanil Kumar, for their wholehearted support during the patient evaluation.

Financial disclosure: None to report.

Informed consent was obtained for this case report.
